# Associations between fears related to safety during sleep and self-reported sleep in men and women living in a low-socioeconomic status setting

**DOI:** 10.1038/s41598-024-54032-w

**Published:** 2024-02-13

**Authors:** Arron T. L. Correia, Philippa E. Forshaw, Laura C. Roden, Gosia Lipinska, H. G. Laurie Rauch, Estelle V. Lambert, Brian T. Layden, Sirimon Reutrakul, Stephanie J. Crowley, Amy Luke, Lara R. Dugas, Dale E. Rae

**Affiliations:** 1 Health Through Physical Activity, Lifestyle and Sport (HPALS) Research Centre and Division of Physiological Sciences, Department of Human Biology, Faculty of Health Sciences, University of Cape Town, Cape Town, 7700 South Africa; 2 Centre for Health and Life Sciences, Coventry University, Coventry, CV1 2DS UK; 3 Department of Psychology, Humanities Faculty, University of Cape Town, Cape Town, South Africa; 4 Division of Endocrinology, Diabetes and Metabolism, University of Illinois Chicago, Chicago, IL USA; 5 Jesse Brown Veterans Affairs Medical Center, Chicago, IL USA; 6 Department of Psychiatry and Behavioral Sciences, Biological Rhythms Research Laboratory, Rush University Medical Center, Chicago, IL USA; 7 Public Health Sciences, Parkinson School of Health Sciences and Public Health, Loyola University Chicago, Maywood, IL USA; 8 Division of Epidemiology and Biostatistics, School of Public Health, Faculty of Health Sciences, University of Cape Town, Cape Town, South Africa

**Keywords:** Personal safety, Sleep environment, Insomnia, Sleep quality, Physiology, Human behaviour

## Abstract

South Africans living in low socioeconomic areas have self-reported unusually long sleep durations (approximately 9–10 h). One hypothesis is that these long durations may be a compensatory response to poor sleep quality as a result of stressful environments. This study aimed to investigate whether fear of not being safe during sleep is associated with markers of sleep quality or duration in men and women. South Africans (n = 411, 25–50 y, 57% women) of African-origin living in an urban township, characterised by high crime and poverty rates, participated in this study. Participants are part of a larger longitudinal cohort study: Modelling the Epidemiologic Transition Study (METS)–Microbiome. Customised questions were used to assess the presence or absence of fears related to feeling safe during sleep, and the Epworth Sleepiness Scale, Pittsburgh Sleep Quality Index (PSQI) and Insomnia Severity Index were used to assess daytime sleepiness, sleep quality and insomnia symptom severity respectively. Adjusted logistic regression models indicated that participants who reported fears related to safety during sleep were more likely to report poor sleep quality (PSQI > 5) compared to participants not reporting such fears and that this relationship was stronger among men than women. This is one of the first studies outside American or European populations to suggest that poor quality sleep is associated with fear of personal safety in low-SES South African adults.

## Introduction

In the US, an adverse social environment, characterised by high disorder, low safety and low social cohesion has been associated with shorter self-reported sleep duration and higher daytime sleepiness^1^. In contrast, we, and others, have previously shown that South Africans of African-origin living in similar adverse communities self-report long, rather than short, time-in-bed (around 8.75 h)^2^ and total sleep times (between 8.5 and 10 h)^3–6^. Previously, individuals reporting higher perceived neighbourhood safety in the South African setting were less likely to report long sleep^6^. Perhaps fears of being unsafe at night or even anxiety related to past traumatic events erode sleep quality, causing individuals to extend their sleep to compensate.

In South Africa, 24% of the population live in urban townships, characterised by temporary housing which provide individuals with little to no security regarding their residence, usually lack basic services and often have buildings which may not comply with government regulations^7^. Khayelitsha, is one such township, in which 55% of residents live in informal dwellings, 38% are unemployed and nearly 75% earn less than R38,200 ($2,680.32) annually^8^. According to the 2020 South African crime statistics, low socioeconomic status (SES) communities like Khayelitsha have the highest number of contact crimes reported, defined as incidents where victims are subjected to violence or threats of violence including murder or attempted murder, sexual offences, and assault or robbery^9^. In particular, Khayelitsha had the fifth highest number of contact crimes nationwide^9^. It seems plausible that individuals living in such a setting may well experience fears related to personal safety, especially at night when trying to sleep.

South African women may be particularly vulnerable to fears related to personal safety given that they are exposed to high levels of gender-based violence. Most sexual offences occur in the home by perpetrators known by the victim, particularly in low SES communities^10,11^. This may translate to women experiencing fears for their safety in their home environments which may further impair their sleep. In fact, Lipinska and Thomas^12^ found that women in South Africa with post-traumatic stress disorder slept better in an unfamiliar bed at the laboratory due to feeling safer than they do at home. There is also a well-established gender disparity in the prevalence of insomnia, with women around 1.25–1.41 times more likely to report insomnia than men^13–15^. Previous research has shown that this gender disparity can largely be explained by socioeconomic factors with the lower SES of women mediating the gender difference in reported sleep problems^16^. Therefore, in a low SES setting, such as Khayelitsha, risk for poor sleep may be higher among women, since a greater percentage of women live in poverty^17^ and are unemployed^18^ compared to men. For these reasons, we split our analyses by gender as it seems likely that there may be barriers to sleep which are greater for women compared to men.

We hypothesise that individuals experiencing fear related to safety during sleep will have poorer sleep and that this relationship will be stronger in women compared to men. Therefore, the aims of this study are to investigate whether fears related to perceptions of safety during sleep are associated with self-reported sleep duration, quality, symptoms of insomnia and daytime sleepiness, and to compare these associations between South Africans men and women living in a low SES neighbourhood.

## Methods

### Study overview and design

This study is a sub-study of a larger study: “Modelling the Epidemiologic Transition Study (METS)—Microbiome”, a prospective longitudinal study investigating associations between the gut microbiota, short chain fatty acids, adiposity and risk for cardiometabolic disease in individuals of African-origin from 5 countries varying by economic development. As protocols for both METS-Microbiome, as well as its parent study, METS,^19,20^ have been previously published, we present only methodological information specific to the current analysis. Data were collected during the baseline METS-Microbiome study (2018–2019). Participants presented to the METS clinic in Khayelitsha, an urban low socioeconomic status informal township in Cape Town. Trained fieldworkers, speaking the home language of the participants, administered questionnaires and made anthropometric measurements. For the South African cohort, METS-Microbiome as well as the current study were approved by the University of Cape Town Faculty of Health Sciences Ethics Committee (HREC Number: 696/2018 and 154/2020) and conducted in accordance with the Declaration of Helsinki^21^. All participants signed informed consent in their home language (English or iXhosa).

### Participants

South African adults of African-origin between the ages of 25 and 50y (n = 411, 57% women), living in Khayelitsha participated in the South African arm of METS-Microbiome. Pregnant or lactating women and individuals with active infections (e.g. HIV, and malaria) were excluded. All participants lived in a combination of formal and semi-formal housing and had been living in Khayelitsha for a minimum of two years. None of the participants in this study were from the same household.

### Questionnaire data

Demographic, medical history and lifestyle information were obtained from the METS-Microbiome questionnaires^19,20^. Participants provided markers of SES status, including highest education level achieved (no formal education, primary school, secondary school, tertiary education), employment status (assessed by the question “Did you do any work for pay in the last month?”), and housing density (assessed by the question “How many people are part of this household, including yourself? The household is defined as people who regularly live and eat together and sleep in the house on at least 4 nights of the week”). Participants indicated whether or not they suffered from any chronic diseases and used any chronic medication. Smoking status was categorized as current smoker, ex-smoker, occasional smoker and non-user (“Never smoked regularly”). Given the small number of individuals in the occasional smokers group, this group was combined with the current smokers group. Current alcohol use (units per week) was also recorded.

Sleep measures included the Epworth Sleepiness Scale (ESS)^22^, the Pittsburgh Sleep Quality Index (PSQI)^23^ and the Insomnia Severity Index (ISI)^24^, which were used to quantify daytime sleepiness, sleep quality and insomnia symptom severity, respectively. The ESS is comprised of 8 questions that are summed to obtain a total score (0–24), with higher scores indicating greater levels of daytime sleepiness^22^. Scores above 10 are indicative of excessive daytime sleepiness. The PSQI contains 19 questions, the scores of which are summed for a global score. A cut-point of > 5 is used to denote poor sleep quality^23^. Bedtime, wake-up time, sleep onset latency and total sleep time (TST) were obtained directly from the PSQI. Time-in-bed (TIB) was calculated as the difference between bedtime and wake-up time reported in the PSQI. The PSQI subcomponent categorical scores for sleep disturbances (None, Slight, Moderate, Severe) and daytime dysfunction (None, Slight, Moderate, Severe) were also used in these analyses. Finally, the ISI is a 7-item questionnaire assessing the nature, severity, and impact of insomnia. It evaluates 7 dimensions over the “last month” using a 5-point Likert scale yielding a total score ranging from 0 to 28. Scores above 14 indicate clinically significant insomnia symptoms^24^. We used a customised questionnaire adapted from the Fear of Sleep Inventory^25^ to investigate perceptions of safety during sleep. Participants were asked to indicate (Yes/No) whether any of the following scenarios normally made it difficult for them to either fall asleep or stay asleep at night: “Fear of not being safe while asleep”, “Fear of being attacked while asleep”, “Fear of falling asleep”, “Being awakened by strange noises out of sleep”, “Dreams about past traumatic experiences” and “Sleeping with the light on to feel safe”. This questionnaire is presented in Supplementary Table S1.

Cronbach’s alpha and factor analyses were done to assess the most appropriate way to analyse the fear of sleep questions. We analysed each question individually as the Cronbach alpha score (alpha = 0.60) was deemed unacceptable, indicating it would be inappropriate to create a composite score from the questions. Therefore, each of the fear items were treated individually as binary variables.

### Anthropometrics

Trained fieldworkers measured the height (m) to the nearest 0.1cm and weight (kg) to the nearest 0.5 kg. Body mass index (BMI) was calculated (kg/m^2^) and participants were classified as normal weight (BMI ≤ 25 kg/m^2^), overweight (BMI > 25 kg/m^2^ and < 30 kg/m^2^) or obese (BMI ≥ 30 kg/m^2^).

### Data and statistical analyses

All data were analysed using Stata (v. 13, StataCorp LLC, College Station, TX, USA) and a value of *p* < 0.05 was deemed statistically significant. Data are presented as mean and standard deviation, median and interquartile range or count (percentage). Normality was assessed using Shapiro Wilk’s test. Between group comparisons were made using independent t-tests, Mann–Whitney U, Chi-squared or Fisher’s Exact tests.

Logistic regression analyses were used to assess associations between self-report measures of TIB, TST, daytime sleepiness, sleep quality, insomnia symptom severity and daytime dysfunction (dependent variables) and the fear-related items (independent variables) stratified by gender. Age was included as a covariate in all analyses a priori and we used univariate analyses to identify other covariates (BMI, smoking status, alcohol consumption, household density, presence of young children, annual household income, employment status and level of education) for each model (Supplementary Tables S3 and S4). Identified covariates with a significance level of *p* < 0.15 were included in each model.

## Results

### Demographic, sleep and fear of sleep characteristics

The descriptive characteristics of the men and women are presented in Table 1. Women had a higher BMI (*p* < 0.001), were less likely to be employed (*p* < 0.001), lived in households with more people (*p* = 0.002), were less likely to be smokers (*p* < 0.001) and consumed less alcohol (*p* < 0.001) than men.

**Table 1:** Descriptive characteristics of participants.

	Women (n = 234)	Men (n = 177)	*p* value
Age (y)	34 (29–42)	36 (31–42)	0.191
BMI (kg m^−2^)	32.71 (26.70–38.21)	21.44 (19.39–24.22)	**< 0.001**
Chronic disease (count, %)	60 (25.6)	37 (20.8)	0.251
Employed (count, %)	69 (29.6)	94 (53.4)	**< 0.001**
Highest degree of formal education (count, %)			0.239
None	2 (0.9)	3 (1.7)	
Primary	142 (60.9)	110 (62.5)	
Secondary	75 (32.2)	47 (26.6)	
Tertiary	14 (6.0)	16 (9.0)	
Household density (people per house)	5 (3–6)	4 (2–5)	**0.002**
Presence of young children (count, %)	187 (79.4)	88 (50.0)	**< 0.001**
Under 2 years old	57 (24.5)*	20 (11.4)*	
Between 3 and 15 years old	171 (73.4)*	83 (47.2)*	
Smoking (count, %)			** < 0.001**
Non-smoker	187 (79.9)*	38 (21.9)*	
Smoker	42 (18.0)*	128 (71.9)*	
Ex-smoker	5 (2.1)*	11 (6.2)*	
Alcohol consumption (standard drinks per week)	0 (0–7)	16 (0–36)	**< 0.001**

*BMI* body mass index. Data are presented as median (interquartile range) or count (percentage). *P* values were determined using Mann–Whitney U and Chi-squared tests. *indicate posthoc differences. Significant values are in bold.

**Figure 1:**
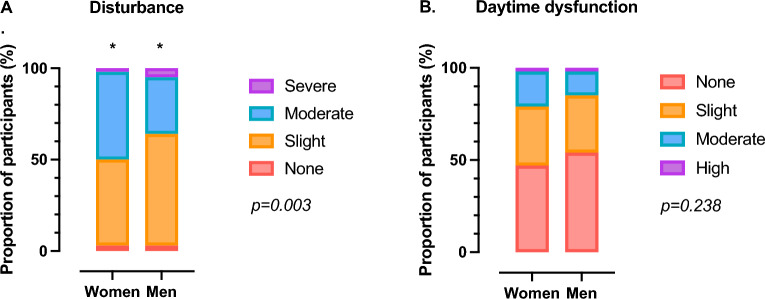
The distribution of scores for the PSQI subcomponents: (**A**) disturbance and (**B**) daytime dysfunction in women (n = 234) and men (n = 177). Chi-squared tests were used to compare distributions between men and women. *indicate posthoc differences between women and men determined using Fisher’s exact.

Self-reported sleep characteristics are presented in Table 2. Mean time-in-bed reported by both men and women was longer than 9 h, with roughly a third of participants reporting poor sleep quality (PSQI > 5) and excessive daytime sleepiness (ESS > 10). Men reported longer sleep onset latencies than women (*p* = 0.013). PSQI sub-component distributions for sleep disturbances and daytime dysfunction are displayed in Fig. 1. The distribution of sleep disturbance severity differed between men and women, with more women reporting moderate (men: 31.3% vs. women: 47.9%, *p* = 0.001) and more men reported slight (men: 61.4% vs. women: 47.0%, *p* = 0.003) sleep disturbances. The distribution of daytime dysfunction related to sleep was similar between men and women, with roughly half reporting some degree of daytime dysfunction (men: 46.0% vs. women: 53.4%, *p* = 0.245).

**Table 2:** Self-reported sleep characteristics of participants.

	Women (n = 234)	Men (n = 177)	*p* value
PSQI bedtime (hh:mm)	21:30 (21:00–22:00)	22:00 (21:00–22:00)	0.579
PSQI wake-up time (hh:mm)	7:00 (6:00–8:00)	7:00 (6:00–8:00)	0.951
PSQI sleep onset latency (min)	20 (10–30)	30 (15–30)	**0.013**
PSQI time-in-bed (h)	9.31 ± 1.55	9.17 ± 1.65	0.376
< 7 h	9 (3.9)	11 (6.3)	0.492
7–9 h	109 (46.8)	84 (47.7)	
> 9 h	115 (49.4)	81 (46.0)	
PSQI total sleep time (h)	8.79 ± 1.53	8.53 ± 1.61	0.138
< 7 h	22 (9.4)	26 (14.7)	0.050
7–9 h	120 (51.3)	95 (53.7)	
> 9 h	92 (39.3)	56 (31.6)	
PSQI score	4 (3–6)	4 (3–6)	0.513
Poor sleep quality (PSQI > 5)	67 (28.6)	53 (30.1)	0.744
ESS score	7 (4–11)	7 (3–10)	0.137
Excessive daytime sleepiness (ESS > 10)	80 (34.3)	49 (27.8)	0.162
ISI score	3 (1–6)	2 (1–5)	0.084
Clinically significant insomnia symptoms (ISI > 14)	13 (5.6)	9 (5.1)	0.834

*ESS* epworth sleepiness scale, *ISI* insomnia severity index, *PSQI* pittsburgh sleep quality index. Data are presented as mean ± standard deviation, median (interquartile range) or count (percentage). *P* values were determined using independent t-tests, Mann–Whitney U and Chi-squared tests. Significant values are in bold.

**Figure 2:**
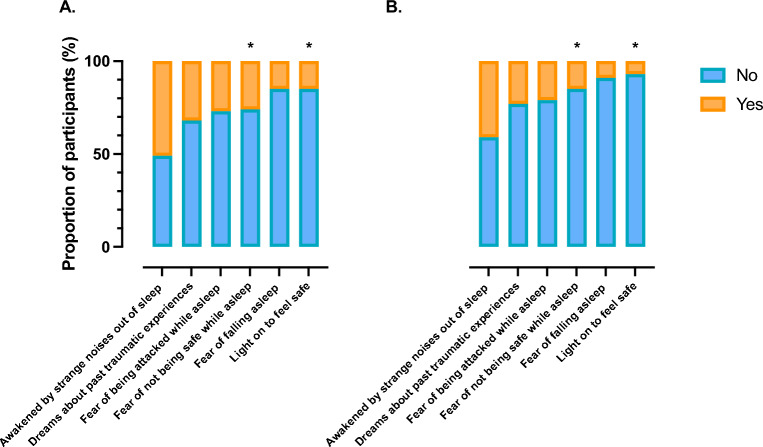
The proportion of (**A**) women (n = 234) and (**B**) men (n = 177) who reported fears related to sleep compared to those who reported no fears related to sleep. Data are presented as frequencies. Chi-squared tests were used to compare distribution differences between men and women. * indicate posthoc differences between women and men determined using Fisher’s exact.

Figure 2 presents participant responses to the fear of sleep questions. Among both men and women, the most common fear response was “Being awakened by strange noises out of sleep”. In addition, more women reported “Fear of not being safe during sleep” (*p* = 0.007) as well as “Sleeping with the light on to feel safe” (*p* = 0.001) than men.

### Associations between fear of sleep and sleep characteristics

#### Men

Among the men, adjusted models show that men who reported “Fear of being attacked during sleep”, “Fear of not being safe during sleep”, “Fear of falling asleep” and “Sleeping with the light on to feel safe” were more likely to report excessive daytime sleepiness than those who did not report fears related to sleep (Table 3). Men who reported any of the fears related to sleep as well as those who were older were more likely to report poor sleep quality. Additionally, “Dreams about past traumatic experiences”, “Fear of not being safe while asleep” or “Fear of falling asleep” along with the covariates alcohol consumption and smoking were associated with increased likelihood of moderate to severe symptoms of insomnia. All fear items (except “Fear of being attacked during sleep”) as well as age, alcohol consumption and smoking were associated with more disturbed sleep. Moderate to severe daytime dysfunction was only associated with the “Fear of falling asleep” fear item. For the time-in-bed models, only age and body mass index showed significant associations with PSQI-derived time-in-bed. Shorter total sleep time was associated with “Fear of not being safe during sleep” and “Sleeping with the light on to feel safe” as well as older age and a higher body mass index.

**Table 3:** Logistic regression model summaries for associations between customised questions around fear related to safety during sleep (independent variable) and self-reported sleep characteristics (dependent variable) in men (n = 177).

Fear items	Excessive daytime sleepiness (ESS > 10)	Poor sleep quality (PSQI > 5)	Clinically significant insomnia symptoms (ISI > 14)	PSQI disturbance item	PSQI daytime dysfunction item	PSQI time-in-bed	PSQI total sleep time
“Awakened by strange noises”	1.46 (0.74–2.88), *p* = 0.278	3.24 (1.56–6.70), ***p* = 0.002**	2.68 (0.56–12.94), *p* = 0.218	1.86 (0.98–3.52), *p* = 0.057	1.24 (0.69–2.21), *p* = 0.476	1.87 (0.99–3.52), *p* = 0.052	1.11 (0.60–2.08), *p* = 0.728
“Dreams about past traumatic experiences”	1.60 (0.74–3.45), *p* = 0.231	5.91 (2.51–13.88), ***p* < 0.001**	6.89 (1.38–34.38), ***p* = 0.019**	2.35 (1.15–4.84), ***p* = 0.020**	1.22 (0.62–2.43), *p* = 0.560	1.25 (0.60–2.60), *p* = 0.969	0.90 (0.45–1.81), *p* = 0.771
“Fear of being attacked during sleep”	2.44 (1.13–5.25), ***p* = 0.023**	3.96 (1.73–9.06), ***p* = 0.001**	2.79 (0.61–12.77), *p* = 0.185	3.38 (1.58–7.22), ***p* = 0.002**	1.23 (0.60–2.50), *p* = 0.570	1.21 (0.57–2.56), *p* = 0.619	0.54 (0.26–1.12), *p* = 0.098
“Fear of not being safe during sleep”	2.73 (1.15–6.47), ***p* = 0.023**	9.09 (3.31–24.99), ***p* < 0.001**	6.50 (1.37–30.87), ***p* = 0.019**	7.19 (2.96–17.47), ***p* < 0.001**	2.20 (0.99–4.90), *p* = 0.054	1.04 (0.44–2.47), *p* = 0.931	0.40 (0.17–0.93), ***p* = 0.033**
“Fear of falling asleep”	3.40 (1.17–9.87), ***p* = 0.024**	6.71 (2.10–21.48), ***p* = 0.001**	13.85 (2.22–86.37), ***p* = 0.005**	5.40 (1.83–15.92), ***p* = 0.002**	4.18 (1.53–11.43), ***p* = 0.005**	1.30 (0.44–3.80), *p* = 0.637	0.41 (0.15–1.13), *p* = 0.085
“Sleeping with the light on to feel safe”	5.00 (1.30–19.27), ***p* = 0.019**	4.90 (1.19–20.20), ***p* = 0.028**	3.42 (0.30–38.89), *p* = 0.322	6.33 (1.63–24.56), ***p* = 0.008**	1.59 (0.45–5.67), *p* = 0.471	0.38 (0.10–1.43), *p* = 0.152	0.17 (0.04–0.64), ***p* = 0.009**

Data are presented as odds ratio (95% confidence interval), *p* value. Logistic regression models were adjusted for age a priori and then further adjusted for covariates which had a *p* value < 0.15 in univariate analyses (Supplementary Table S3). Additional covariates were as follows: for excessive daytime sleepiness model: body mass index (BMI); for poor sleep quality model: annual household income and level of education; for insomnia symptoms model: smoking, alcohol consumption, presence of young children and household density; for sleep disturbances model: BMI, smoking and alcohol consumption; for the time-in-bed models: employment status and level of education and for total sleep time models: BMI, employment status, smoking and level of education. Significant values are in bold.

#### Women

Fully adjusted models also show that women who reported fears related to sleep (with the exception of “Sleeping with the light on to feel safe”) were more likely to report excessive daytime sleepiness and poor sleep quality than those not reporting any fear related to sleep (Table 4). All fear items (with the exception of “Dreams about past traumatic events” and “Fear of being attacked during sleep”) as well as age were associated with increased likelihood of moderate to severe symptoms of insomnia. Unlike in the men, only one fear item, “Dreams about past traumatic events”, was associated with more disturbed sleep. Finally, while women who report “Fear of not being safe while asleep” and “Fear of being attacked while asleep” were more likely to report longer time-in-bed, those who were employed were less likely to report long time-in-bed. In the models for both time-in-bed and total sleep time, a higher BMI was significantly associated with longer time-in-bed and shorter sleep respectively.

**Table 4:** Logistic regression model summaries for associations between customised questions around fear related to safety during sleep (independent variable) and self-reported sleep characteristics (dependent variable) in women (n = 234).

Fear item	Excessive daytime sleepiness (ESS > 10)	Poor sleep quality (PSQI > 5)	Clinically significant insomnia symptoms (ISI > 14)	PSQI disturbance item	PSQI daytime dysfunction item	PSQI time-in-bed	PSQI total sleep time
“Awakened by strange noises”	2.62 (1.45–4.72), ***p* = 0.001**	3.45 (1.82–6.55), ***p* < 0.001**	4.98 (1.04–23.86), ***p* = 0.045**	1.53 (0.92–2.55), *p* = 0.105	1.00 (0.61–1.62), *p* = 0.988	1.36 (0.79–2.33), *p* = 0.261	0.91 (0.54–1.54), *p* = 0.735
“Dreams about past traumatic experiences”	2.05 (1.12–3.74), ***p* = 0.020**	2.18 (1.18–4.05), ***p* = 0.013**	2.44 (0.75–7.97), *p* = 0.140	2.11 (1.21–3.70), ***p* = 0.009**	1.15 (0.68–1.93), *p* = 0.612	1.67 (0.94–2.99), *p* = 0.081	1.13 (0.65–1.99), *p* = 0.661
“Fear of being attacked during sleep”	3.42 (1.81–6.43), ***p* < 0.001**	2.13 (1.12–4.03), ***p* = 0.020**	2.30 (0.70–7.58), *p* = 0.170	1.28 (0.72–2.28), *p* = 0.408	1.15 (0.66–1.98), *p* = 0.627	2.01 (1.12–3.91), ***p* = 0.021**	1.58 (0.86–2.88), *p* = 0.138
“Fear of not being safe during sleep”	2.43 (1.29–4.57), ***p* = 0.006**	3.61 (1.80–6.54), ***p* < 0.001**	3.77 (1.14–12.48), ***p* = 0.030**	1.30 (0.72–2.33), *p* = 0.375	1.29 (0.74–2.25), *p* = 0.365	2.16 (1.14–4.09), ***p* = 0.018**	1.31 (0.71–2.40), *p* = 0.382
“Fear of falling asleep”	3.44 (1.13–5.28), ***p* = 0.023**	3.30 (1.53–7.12), ***p* = 0.002**	3.74 (1.04–13.44), ***p* = 0.043**	1.38 (0.67–2.82), *p* = 0.384	1.25 (0.66–2.37), *p* = 0.488	1.76 (0.82–3.77), *p* = 0.147	0.94 (0.45–1.97), *p* = 0.865
“Sleeping with the light on to feel safe”	1.91 (0.91–4.00), *p* = 0.086	1.57 (0.73–3.37), *p* = 0.246	5.92 (1.54–22.77), ***p* = 0.010**	0.80 (0.40–1.60), *p* = 0.530	0.95 (0.49–1.85), *p* = 0.890	1.11 (0.54–2.29), *p* = 0.776	1.08 (0.54–2.15), *p* = 0.820

Data are presented as odds ratio (95% confidence interval), *p* value. Logistic regression models were adjusted for age a priori and then adjusted for covariates which had a *p* value < 0.15 in univariate analyses (Supplementary Table S4). Additional covariates were as follows: for excessive daytime sleepiness model: smoking, alcohol consumption, household density and level of education; for poor sleep quality model: alcohol consumption per week, level of education and presence of young children in the house; for insomnia symptoms model: alcohol consumption and level of education; for the time-in-bed models: BMI, employment status, presence of young children and household density and for the total sleep time models: BMI and employment status. Significant values are in bold.

## Discussion

We present sleep quality data from the METS-Microbiome study, in which we explored the relationship between sleep-related fear and self-reported measures of sleep duration and quality as well as daytime sleepiness and dysfunction among South African adults living in a low SES environment. The most commonly reported sleep-related fear item was being awakened by strange noises, followed by dreams of past traumatic experiences, fear of being attacked while asleep and then fear of not being safe while asleep. Our study confirms previous findings indicating longer sleep time and time-in-bed among low-SES South Africans^2–6^. Notably, almost half of our participants spent more than 9h in bed. Accompanying the observed long time-in-bed, approximately half the participants reporting some degree of sleep disturbance and daytime dysfunction related to sleep, and about one third reporting excessive daytime sleepiness. Thus, despite self-reported long sleep opportunities, participants report disturbed sleep, which in turn may affect their daytime function.

One might hypothesize that in the context of these low SES adults, longer sleep opportunities may be to compensate for poor sleep quality, driven at least partially, by fear for their safety during sleep. Our findings confirm that there is an inverse association between sleep-related fear and sleep quality and that women who reported feeling unsafe during sleep were more likely to report longer sleep opportunities (time-in-bed > 9 h) but without corresponding longer total sleep times. Furthermore, participants who answered “Yes” to any of the fear-related sleep items were more likely to report more daytime sleepiness, poorer sleep quality and more insomnia-type symptoms than those who feel safe during sleep. The latter finding is similar to previous work by Mellman et al.^26^ which showed that fear of sleep was correlated with insomnia severity in urban African-Americans living in the US. In contrast, however, we did not observe any association between shorter sleep and fear among the METS participants. This may be because METS participants compensated with long sleep opportunities, with very few being classified as short sleepers based either on time-in-bed or total sleep time. Alternatively, the high unemployment rate, leading to a lack of work obligations and subsequently more free time but less expendable income for entertainment, combined with a lack of infrastructure, may help explain why longer sleep durations are only observed in this population but not in urban African-Americans in the US. Thus, in the South African context, fears regarding safety at night appear to reduce sleep quality, despite a seemingly adequate sleep opportunity. Fears related to safety during sleep may lead to hypervigilance, increasing sleep disturbances and reducing overall sleep quality. This idea is supported by the same study mentioned above which showed that in a US urban, low SES environment, higher fear of sleep scores correlated with markers of autonomic dysregulation during sleep^26^.

While the focus of this paper was to explore associations between fear and self-reported sleep parameters, we also observed some of the expected relationships between sleep and covariates, such as age, alcohol consumption, smoking status and BMI^27–30^. Specifically, among the men, independent of the fear items, an older age was associated with poorer sleep quality scores and more disturbed sleep, participants who used alcohol or smoked were more likely to report moderate to severe insomnia symptoms, and older participants as well as those with higher BMI’s were more likely to report shorter sleep durations. In contrast, among the women older age was associated with increased likelihood of moderate to severe symptoms of insomnia and those with a higher BMI were more likely to report longer time-in-bed but shorter total sleep time, independent of the fear items. Compared to the fear-related items, however, the contributions of these covariates were small which gives us confidence that over and above these well-documented relationships^27–30^, the fear items are still independently related to, and give some additional explanations for, poor or short sleep.

Between men and women we saw both similarities and differences in the associations between fear of sleep and sleep parameters. Answering “Yes” to any of the fear-related items (except “Sleeping with the light on to feel safe”) was associated with increased odds for poor quality sleep in both men and women. In women, however, sleep-related fears were also consistently associated with increased odds for excessive daytime sleepiness, while in men the associations were consistently associated with increased risk for more disturbed sleep. While both men and women demonstrated associations between fears related to safety and markers of poor sleep, the associations were stronger among the men, especially for poor sleep quality and disturbed sleep, which was contrary to our hypothesis. This is in contrast to a previous study which found women were more vulnerable to fear-based sleep disruptions and that their autonomic nervous system functioning during sleep may be more sensitive to environmental stress, especially when that stress was related to exposure to violence^26^. There are several possible reasons underlying our observations. Firstly, it may be linked to methodological differences as the above study by Mellman et al.^26^ used objective sleep measures whereas this study utilized subjective sleep measures and we investigated the effect of presence or absence of fears related to sleep as opposed to severity of fear symptoms. Alternatively, given South Africa’s high prevalence of gender-based violence it is possible that women in this community frequently feel vulnerable and thus potentially have become desensitized to fears related to safety. One might hypothesise that although the level of fear experienced by men and women does not appear to be different during sleep, sleep is disrupted to a lesser extent in the women because of a blunted emotional and/or physiological response to fear-related stimuli. Therefore, they may have a higher threshold for fear-related responses to affect their sleep. Finally, it may be that men feel a societal responsibility to protect their family at night causing a state of alertness to threats which results in a state of hyperarousal unconducive to sleep^34^.

Finally, this is one of the few studies confirming the relationship between perceptions of safety and sleep outcomes in populations outside of American or European populations. Notably it is the first study to investigate the association between sleep-related fear and sleep quality, daytime sleepiness, and insomnia in a South African African-origin population. We are aware of only two other studies which have investigated the associations between fear of sleep and insomnia^26,35^. Our study, therefore, extends that research by also including measures of sleep quality and daytime sleepiness. Previous work, including one study in South Africa among 3854 older (60 ± 12 y) adults, has largely looked at associations between perceived neighbourhood safety and self-reported sleep quality^6,36^ whereas we investigated the association between people’s feelings of safety in their homes prior to and during sleep with self-reported sleep measures. Although Poindexter et al.^37^ found that perceptions of the neighbourhood environment were linked to fear of sleep, Simonelli et al.^38^ found that after accounting for feelings of home safety, neighbourhood safety did not impact sleep among adults living in Argentina. These findings indicate that perceptions of the safety of an individual’s immediate sleep environment may be more relevant than their neighbourhood safety. Additionally, this is the first study to investigate how the relationship between perceived safety and sleep quality varies between men and women. Our research highlights the importance of improving community and home safety to allow for better sleep quality, rather than sleep duration, which may have knock-on effects on physical and mental health. Improving sleeping conditions may provide one opportunity of breaking the vicious cycle of poverty and poor health.

Our study is not without limitations, including the exclusive use of self-report data for all the sleep measures. As self-reported total sleep time has consistently been shown to overestimate measured sleep duration^39^, the problem may be even more significant than what we have found in this study. Furthermore, the binary “yes/no” outcomes of the fear of sleep questions as opposed to Likert scale answers may limit the sensitivity of this measurement. The binary nature of these variables also limited our ability to conduct a mediation analysis to directly test the relationship between fears related to safety during sleep, sleep quality and long time-in-bed. Additionally, there may be other variables which were not investigated in this study, such as childcare or other societal responsibilities, length of residence in the community, sleep disorders, psychiatric disorders or measures of distress, all of which may influence both the association between fear and sleep and the differences observed between men and women. Thus, qualitative research as well as more detailed demographic, sleep and psychiatric questionnaires in future studies are recommended to provide further insights. Future research including physiological measures such as cortisol level assessment or heart rate variability may also be useful to elucidate whether autonomic dysregulation does underpin the association between fear-related stimuli and markers of poorer sleep. Finally, we acknowledge the cross-sectional study design which prevents us from inferring causality. It may be that poor sleep quality heightens feelings of vulnerability or fear as opposed to the other way around. Subsequent studies should utilise longitudinal data to draw more definitive conclusions on the extent to which fear of sleep impairs sleep quality.

## Conclusion

We present some of the first data outside of the European and American settings showing that African-origin South Africans adults living in a low-SES, high-crime environment who report fears related to feeling unsafe during sleep experience low-quality sleep accompanied with daytime sleepiness and dysfunction. This has long-term implications for physical and mental health outcomes in participants, already experiencing reduced access to health care as a result of the low-SES setting^40–44^. Further research measuring habitual sleep characteristics, including objectively measured sleep behaviour, and more detailed data on psychiatric symptoms, such as past trauma, depression and anxiety, especially as they relate to nocturnal fears around sleep, are warranted. Additionally, further unpacking the sexual dimorphism in the experience and perception of fears related to safety during sleep is necessary.

### Supplementary Information

Supplementary Tables.

## Data Availability

The datasets generated during and/or analysed during the current study are available from the corresponding author on reasonable request.
