# Supplementary material for: Associations between fears related to safety during sleep and self-reported sleep in men and women living in a low-socioeconomic status setting
Source: Sci Rep. 2024 Feb 13;14:3609. doi: 10.1038/s41598-024-54032-w (PMC10864334; doi:10.1038/s41598-024-54032-w)
Supplement: Supplementary file 1 — Supplementary Tables. [file 41598_2024_54032_MOESM1_ESM.docx]

Table S1: Customised questionnaire adapted from the Fear of Sleep Inventory used to asses fears related to safety during sleep.

Do you have any difficulty falling asleep or staying asleep for a long time normally?

 Yes  No

If so, why? (tick where appropriate). You may tick more than one option.

|  |  | Tick |
| --- | --- | --- |
| a) | Fear of not being safe while asleep |  |
| b) | Fear of being attacked while asleep |  |
| c) | Fear of falling asleep |  |
| d) | Being awakened by strange noises out of sleep |  |
| e) | Dreams about past traumatic experiences |  |
| f) | Sleeping with the light on to feel safe |  |

Table S2: Correlations between the various fear-related questions.

|  | Fear of not being safe while asleep | Fear of being attacked while asleep | Fear of falling asleep | Being awakened by strange noises out of sleep | Dreams about past traumatic experiences | Sleeping with the light on to feel safe |
| --- | --- | --- | --- | --- | --- | --- |
| Fear of not being safe while asleep | 1.000 |  |  |  |  |  |
| Fear of being attacked while asleep | **0.8010^***^** | 1.000 |  |  |  |  |
| Fear of falling asleep | **05680^***^** | **0.5815^***^** | 1.000 |  |  |  |
| Being awakened by strange noises out of sleep | **0.4791^***^** | **0.5236**^***^ | **0.3291^***^** | 1.000 |  |  |
| Dreams about past traumatic experiences | **0.4647^***^** | **0.4592^***^** | **0.2907^***^** | **0.4829^***^** | 1.000 |  |
| Sleeping with the light on to feel safe | **0.2406^***^** | **0.2019^***^** | **0.1153^***^** | **0.3134^***^** | **0.1606**^**^ | 1.000 |

*^*^ p-value < 0.05, ^**^ p-value < 0.01, ^***^ p-value < 0.001*

Table S3: Univariate analyses between each of the potential covariates and self-reported excessive daytime sleepiness as measured by Epworth Sleepiness Scale, poor sleep quality as measured by Pittsburgh Sleep Quality Index, clinically significant symptoms of insomnia as measured by Insomnia Severity Index, sleep disturbances as measured by the Pittsburgh Sleep Quality Index and daytime dysfunction as measured by Pittsburgh Sleep Quality Index as well as self-reported time-in-bed and total sleep time in men (n=177).

|  | **Excessive daytime sleepiness (ESS>10)** | **Poor sleep quality (PSQI>5)** | **Clinically significant insomnia symptoms (ISI>14)** | **Disturbance** | **Daytime dysfunction** | **Time-in-bed** | **Total sleep time** |
| --- | --- | --- | --- | --- | --- | --- | --- |
| Age | 0.94 (0.94-1.03), p=0.505 | 1.06 (1.02-1.12), **p-0.006** | 1.03 (0.94-1.13), p=0.500 | 1.05 (1.01-1.09), **p=0.026** | 1.01 (0.97-1.05), p=0.724 | 0.96 (0.92-1.00), **p=0.038** | 0.96 (0.92-1.00), **p<0.031** |
| Body mass index | 1.06 (0.98-1.15), p=0.115 | 1.03 (0.96-1.12), p=0.392 | 1.02 (0.88-1.19), p=0.769 | 1.06 (0.99-1.14), **p=0.118** | 1.02 (0.95-1.09), p=0.587 | 0.96 (0.90-1.03), p=0.301 | 0.91 (0.84-0.98), p=0.013 |
| Smoking | 1.23 (0.78-1.94), p=0.369 | 0.88 (0.55-1.41), p=0.592 | 0.33 (0.09-1.13), p=0.078 | 1.44 (0.94-2.20), p=0.095 | 1.08 (0.72-1.63), p=0.714 | 1.23 (0.82-1.85), p=0.326 | 1.45 (0.96-2.19), p=0.075 |
| Alcohol consumption | 1.00 (0.99-1.01), p=0.964 | 1.00 (0.99-1.02), p=0.528 | 1.02 (1.00-1.03), p=0.065 | 1.01 (1.00-1.02), p=0.057 | 1.00 (0.99-1.01), p=0.925 | 1.01 (1.00-1.02), p=0.170 | 1.00 (0.99-1.01), p=0.428 |
| Household density | 0.98 (0.85-1.13), p=0.821 | 0.96 (0.84-1.11), p=0.606 | 0.65 (0.43-1.00), p=0.052 | 0.99 (0.88-1.13), p=0.931 | 1.01 (0.90-1.15), p=0.832 | 0.93 (0.82-1.04), p=0.209 | 0.96 (0.85-1.08), p=0.507 |
| Presence of young children | 0.83 (0.43-1.60), p=0.581 | 0.93 (0.49-1.78), p=0.830 | 0.11 (0.01-0.94), **p=0.044** | 0.82 (0.45-1.49), p=0.512 | 0.86 (0.49-1.52), p=0.603 | 0.90 (0.51-1.60), p=0.724 | 0.99 (0.56-1.75), p=0.980 |
| Work for pay | 0.89 (0.46-1.72), p=0.726 | 1.22 (0.64-2.34), p=0.546 | 1.80 (0.43-7.42), p=0.419 | 1.26 (0.69-2.29), p=0.454 | 1.49 (0.84-2.65), p=0.172 | 0.49 (0.27-0.88), **p=0.017** | 0.58 (0.32-1.03), p=0.064 |
| Annual household income | 1.06 (0.85-1.32), p=0.624 | 0.83 (0.66-1.05), p=0.122 | 0.87 (0.54-1.42), p=0.583 | 0.89 (0.73-1.09), p=0.256 | 1.06 (0.88-1.27), p=0.571 | 0.91 (0.75-1.11), p=0.356 | 0.93 (0.77-1.13), p=0.468 |
| Level of education | 1.04 (0.69-1.57), p=0.841 | 1.53 (1.02-2.32), **p=0.042** | 1.10 (0.48-2.50), p=0.824 | 1.28 (0.88-1.87), p=0.194 | 1.24 (0.87-1.77), p=0.228 | 0.69 (0.48-0.99), **p=0.043** | 0.70 (0.49-1.00), p=0.051 |

Table S4: Univariate analyses between each of the potential covariates and self-reported excessive daytime sleepiness as measured by Epworth Sleepiness Scale, poor sleep quality as measured by Pittsburgh Sleep Quality Index, clinically significant symptoms of insomnia as measured by Insomnia Severity Index, sleep disturbances as measured by the Pittsburgh Sleep Quality Index and daytime dysfunction as measured by Pittsburgh Sleep Quality Index as well as self-reported time-in-bed and total sleep time in women (n=234).

|  | **Excessive daytime sleepiness (ESS>10)** | **Poor sleep quality (PSQI>5)** | **Clinically significant insomnia symptoms (ISI>14)** | **Disturbance** | **Daytime dysfunction** | **Time-in-bed** | **Total sleep time** |
| --- | --- | --- | --- | --- | --- | --- | --- |
| Age | 1.00 (0.97-1.04), p=0.895 | 1.05 (1.01-1.08), **p=0.014** | 1.14 (1.06-1.24), **p=0.001** | 1.03 (1.00-1.06), **p=0.071** | 1.02 (0.99-1.05), p=0.152 | 0.96 (0.93-1.00), **p=0.025** | 0.93 (0.90-0.96), **p<0.001** |
| Body mass index | 1.01 (0.98-1.04), p=0.514 | 1.01 (0.97-1.04), p=0.720 | 1.01 (0.94-1.08), p=0.857 | 1.01 (0.98-1.04), **p=0.579** | 1.01 (0.98-1.04), p=0.384 | 0.95 (0.92-0.98), **p=0.001** | 0.96 (0.93-0.99), **p=0.010** |
| Smoking | 1.73 (1.16-2.58), **p=0.008** | 0.91 (0.56-1.40), p=0.666 | 0.83 (0.32-2.16), p=0.711 | 0.94 (0.65-1.35), p=0.725 | 0.92 (0.66-1.33), p=0.651 | 1.25 (0.85-1.83), p=0.257 | 1.07 (0.74-1.55), p=0.725 |
| Alcohol consumption | 1.02 (1.00-1.04), p=0.052 | 1.02 (1.00-1.03), p=0.123 | 1.01 (0.99-1.02), p=0.073 | 0.98 (0.92-1.034), p=0.435 | 1.01 (0.99-1.02), p=0.463 | 1.01 (0.99-1.02), p=0.392 | 1.01 (0.99-1.02), p=0.392 |
| Household density | 0.87 (0.75-1.00), p=0.051 | 0.96 (0.83-1.11), p=0.597 | 0.98 (0.74-1.30), p=0.882 | 0.98 (0.86-1.11), p=0.704 | 0.98 (0.87-1.11), p=0.769 | 0.83 (0.73-0.95), **p=0.007** | 0.94 (0.82-1.06), p=0.301 |
| Presence of young children | 0.85 (0.44-1.64), p=0.622 | 0.60 (0.31-1.17), p=0.135 | 1.45 (0.31-6.79), p=0.634 | 0.87 (0.47-1.60), p=0.649 | 0.95 (0.52-1.74), p=0.879 | 0.47 (0.24-0.91), **p=0.025** | 0.69 (0.37-1.29), p=0.242 |
| Work for pay | 0.96 (0.53-1.74), p=0.892 | 1.12 (0.61-2.08), p=0.713 | 1.06 (0.32-3.56), p=0.925 | 0.92 (0.53-1.58), p=0.751 | 1.44 (0.86-2.40), p=0.166 | 0.48 (0.27-0.85), **p=0.012** | 0.55 (0.31-0.97), **p=0.038** |
| Annual household income | 0.72 (0.59-0.87), **p=0.001** | 1.02 (0.86-1.21), p=0.823 | 0.78 (0.52-1.17), p=0.231 | 0.91 (0.78-1.06), p=0.238 | 1.00 (0.86-1.15), p=0.961 | 1.01 (0.86-1.17), p=0.946 | 1.04 (0.90-1.21), p=0.583 |
| Level of education | 0.74 (0.51-1.08), p=0.114 | 0.64 (0.42-0.97), **p=0.036** | 0.40 (0.17-0.93), **p=0.034** | 0.81 (0.59-1.14), p=0.238 | 0.96 (0.70-1.30), p=0.771 | 0.82 (0.58-1.15), p=0.258 | 1.04 (0.75-1.44), p=0.821 |
